# High-Resolution Melting Curve Analysis, a Rapid and Affordable Method for Mutation Analysis in Childhood Acute Myeloid Leukemia

**DOI:** 10.3389/fped.2014.00096

**Published:** 2014-09-09

**Authors:** Yin Liu, Jingyan Tang, Peter Wakamatsu, Huiliang Xue, Jing Chen, Paul S. Gaynon, Shuhong Shen, Weili Sun

**Affiliations:** ^1^Department of Hematology/Oncology, Shanghai Children’s Medical Center, Shanghai Jiaotong University School of Medicine, Shanghai, China; ^2^Division of Hematology, Oncology and Blood and Marrow Transplantation, Children’s Center for Cancer and Blood Disease, Children’s Hospital Los Angeles, Keck School of Medicine, University of Southern California, Los Angeles, CA, USA

**Keywords:** *FLT3*, *NPM1*, *MLL*, high-resolution melting curve analysis, acute myeloid leukemia, childhood

## Abstract

**Background:** Molecular genetic alterations with prognostic significance have been described in childhood acute myeloid leukemia (AML). The aim of this study was to establish cost-effective techniques to detect mutations of FMS-like tyrosine kinase 3 (*FLT*3), nucleophosmin 1 (*NPM1*), and a partial tandem duplication within the mixed-lineage leukemia (*MLL-PTD*) genes in childhood AML.

**Procedure:** Ninety-nine children with newly diagnosed AML were included in this study. We developed a fluorescent dye SYTO-82 based high-resolution melting (HRM) curve analysis to detect *FLT*3 internal tandem duplication (*FLT3-ITD*), *FLT*3 tyrosine kinase domain (*FLT3-TKD*), and *NPM1* mutations. *MLL-PTD* was screened by real-time quantitative PCR.

**Results:** The HRM methodology correlated well with gold standard Sanger sequencing with less cost. Among the 99 patients studied, the *FLT3-ITD* mutation was associated with significantly worse event-free survival (EFS). Patients with the *NPM1* mutation had significantly better EFS and overall survival. However, HRM was not sensitive enough for minimal residual disease monitoring.

**Conclusion:** High-resolution melting was a rapid and efficient method for screening of *FLT*3 and *NPM1* gene mutations. It was both affordable and accurate, especially in resource underprivileged regions. Our results indicated that HRM could be a useful clinical tool for rapid and cost-effective screening of the *FLT3* and *NPM1* mutations in AML patients.

## Introduction

Acute myeloid leukemia (AML) is a group of heterogeneous disease harboring different genetic alterations and with considerable diversity in clinical behavior and prognosis (1–3). Karyotype is the major prognostic factors in AML (1–3). Several molecular alterations have been previously identified in children with AML, especially in cases with cytogenetic normal AML (CN-AML) (4–15).

FMS-like tyrosine kinase receptor (*FLT3*) internal tandem duplications (ITDs) are caused by the duplication of the exon 14 sequence at juxtamembrane domain, leading to constitutive activation of downstream signaling (16, 17). *FLT3-ITD* has been described in 4–27% of patients with childhood AML and a high *FLT3-ITD* allelic ratio is associated with worse outcome (4–7). Point mutations in codon 835 or 836 of the *FLT3* gene tyrosine kinase domain (*FLT3-TKD*) occur in 3–11% of patients (4–7, 18, 19), but the prognostic relevance remains to be defined.

Nucleophosmin 1 (*NPM1*) is a nuclear and cytoplasmic protein, which functions as a molecular chaperone to prevent protein aggregation in the nucleolus and to regulate p53 levels (9). Point mutations within the C-terminal region of the *NPM1* gene result in cytoplasmic accumulation of the mutated NPM protein (9, 11, 12). This occurs in ~25% of pediatric CN-AML cases and is associated with a favorable clinical outcome (8, 10).

Partial tandem duplication (*PTD*) of the myeloid/lymphoid or mixed-lineage leukemia (*MLL*) gene without major chromosome structural aberrations involving band 11q23 has been identified in 5–13% of patients with *de novo* AML (13–15). Event-free survival (EFS) and complete remission (CR) duration were shorter in adult patients with CN-AML bearing *MLL-PTD* mutation than patients with the wild-type MLL gene (13–15).

In the United States and Europe, mutation detections of *FLT3-ITD*, *FLT3-TKD*, and *NPM1* have been established as conventional diagnostic tests in clinical practice. However, they have not been applied to routine clinical care in China. Part of the reason has been cost (20, 21); when facing a catastrophic event such as cancer, it remains a significant challenge for Chinese pediatric oncologists to provide valuable and affordable clinical diagnostic tests.

Traditionally, *FLT3-ITD*, *FLT3-TKD*, and *NPM1* mutations are detected by PCR followed by electrophoresis or Taqman RQ-PCR (17, 22–24). The traditional PCR technique is convenient and less expensive. However, it is an open tube system with increased possibility of false positive results due to amplification product carry-over contamination. Taqman RQ-PCR is a closed-tube system requiring no post-PCR processing, and it has increased sensitivity and higher resolution when compared to traditional PCR. However, this technique can only detect known mutations matching the designed probe. Thus, the search continues for a sensitive, reliable, and good manufacturing procedure (GMP) leveled detection system for both the known and potential unknown gene mutations in clinical diagnosis.

High-resolution melting (HRM) curve analysis is a homogenous, closed-tube, post-PCR technique for rapidly and efficiently discovering genetic variations in DNA fragments (25–27), based on the sequence dependent dissociation behavior of DNA when exposed to increasing temperature. It is a simple, powerful, and cost-effective method for a large scale genotyping project (28). The accuracy of the technique depends on appropriate PCR design and saturating DNA dyes.

In this study, we investigated using HRM analysis to evaluate the prevalence of *FLT3-ITD*, *FTL3-TKD*, and *NPM1* in childhood AML. We also developed real-time quantitative PCR (RQ-PCR) methodology to detect *MLL-PTD* gene mutations. We validated the HRM technology and analyzed the clinical significance of gene mutations in a group of 99 children with AML who were enrolled in the AML-XH-99 protocol. The AML-XH-99 protocol was a single arm clinical study to examine homoharringtonine, a plant alkaloid that has been used to treat leukemia in China since the 1980s (29), in combination with chemotherapy to treat childhood AML. The outcome of this trial has been previously published (30).

## Materials and Methods

### Patients

From January 1999 to December 2008, a total of 99 newly diagnosed PML/RARA negative AML pediatric patients were included in this study. All 99 patients were already enrolled in the AML-XH-99 protocol and had available bone marrow (BM) samples frozen in the Biobank at Shanghai Children’s Medical Center.

At diagnosis, all patients underwent blood testing for complete blood counts and biochemistry panels. BM cells were aspirated for morphology, immunohistochemistry, karyotype, immunophenotyping, and molecular testings for RUNX1-RUNX1T1(AML1-ETO), CBFB-MYH11, or PML-RARA gene mutations (31). CN-AML is defined as leukemia without chromosomal abnormalities by karyotype and negative for RUNX1-RUNX1T1, CBFB-MYH11, or PML-RARA by PCR.

Additional BM samples were collected for research purposes at the time of diagnosis (after a legal guardian signed an informed consent). The mononuclear cells were isolated by Ficoll-Hypaque gradient and cryopreserved in the Biobank. This study was approved by the Institutional Review Board of Shanghai Children’s Medica Center.

### Chemotherapy

All patients were treated on the AML-XH-99 protocol, which included induction, consolidation, and continuation therapies (30). Induction consisted two cycles of DAE (daunorubicin 40 mg/m^2^/day, days 1–3, cytarabine 200 mg/m^2^ every 12 h, days 1–7, and etoposide 100 mg/m^2^/day, days 1–3). Consolidation included two courses of chemotherapy: (A) DA (cytarabine 2 g/m^2^ every 12 h, days 1–3, and daunorubicin 30 mg/m^2^/day, day 1 and 2); and (B) EA (cytarabine 2 g/m^2^ every 12 h, days 1–3, and VP-16 160 mg/m^2^/day, day 1 and 2). For continuation therapy, DA alternating with EA, followed by two courses of HA (homoharringtonine 3–4 mg/m^2^/day, days 1–9, and cytarabine 75 mg/m^2^ every 12 h, days 1–7), were given for a total of 12 cycles (30).

Complete remission was defined as marrow blasts <5% with evidence of blood count recovery. Central nervous system leukemia prevention consisted of intrathecal (IT) chemotherapy with dexamethasone 5 mg/m^2^, methotrexate 12.5 mg/m^2^ (maximum 12.5 mg), and cytarabine 1 mg/kg (maximum 35 mg) at various time points (30). Allogeneic hematopoietic stem cell transplantation (Allo-HSCT) was performed in some relapsed or refractory patients with suitable donors who had achieved CR or at least a good partial response (PR, defined as a BM blast reduction of >50%) after salvage chemotherapy.

### Samples

In this study, BM samples at diagnosis were analyzed for gene mutations. Control BM samples came from patients with idiopathic thrombocytopenia who underwent BM aspiration to rule out malignancy.

Total RNA was extracted from 1 × 10^7^ BM mononuclear cells (BMMC) using the phenol–chloroform–isopropanol method (TRIzol^®^, Invitrogen). One microgram of total RNA was reverse transcribed according to the manufacturer’s protocols (Takara, Japan). RNA quality and quantity were assessed relative to the GAPDH housekeeping gene on the LightCycler^®^ 480 System (Roche Diagnostics, Penzberg, Germany). Samples with Ct_GAPDH_ values over 30 were considered uninterpretable.

### High-resolution melting analysis of *FLT3-ITD*, *FLT3-TKD*, and *NPM1* mutation

The PCR and melting analysis for *FLT3* and *NPM1* mutations was performed on the LightCycler^®^ 480, a real-time PCR machine with HRM and 96/384 well capacity. All samples were tested in triplicates. One positive control and one negative control with each gene mutation, as well as one blank control with distilled water only, were included in triplets on each run of unknowns. PCR was performed from ~10 ng of cDNA, using 0.3 μM each of the relevant forward and reverse primers in a total volume of 10 μl containing 5 μl ES Taq HS premix (Takara) and 5 μM SYTO-82 (Invitrogen) (32). PCR was performed at 94°C for 2 min, followed by 45 cycles at 94°C for 10 s, 56°C for 10 s, 72°C for 20 s, and a final extension step at 72°C for 5 min. The cycling conditions were the same for all three amplicons, allowing them to be performed in one run. The program ran for 40 cycles at 95°C for 30 s, 40°C for 30 s, then 60°C −95°C (5 s, 1°C/s). Upon completion of the run, analysis was performed using the software supplied with the LightCycler^®^ 480. The melting curves were normalized, and temperature shifted to allow samples to be directly compared. Difference plots were generated by selecting a negative control as the baseline. The fluorescence of all other samples was plotted relative to this sample. Significant differences in fluorescence were indicative of mutations.

The primers used were: (1) *FLT3-ITD*: exon 11 (ITD-F) GCAATTTAGGTATGAAAGCCAGC, exon 15 (ITD-R) GTTGCGTTCATCACTTTTCCAA; (2) *FLT3-TKD*: TKD-F ACGTGCTTGTCACCC, TKD-R TTAATGGTGTAGATGCCTTCAAAC; and (3) *NPM1*: NPM-F TGACTGACCAAGAGGCTATTC, NPM-R GACAGCCAGATATCAACTGTTAC.

### RQ-PCR analysis of *MLL-PTD* mutation

Real-time quantitative PCRs were performed on the LightCycler^®^ 480 using ~20 ng of cDNA and 0.5 μM each of the relevant forward and reverse primers: MLL-F3: AAGCAGCCTCCACCACCAGAAT and MLL-R3: CCACGAGGTTTTCGAGGACTA in a total volume of 10 μl containing with 5 μl SYBR Green I premix (Takara) at 94°C for 2 min, followed by 35 cycles at 94°C for 10 s, 56°C for 20 s. Samples with CT values below 35 and with satisfying melting peak at 82–84°C were considered as *MLL-PTD* mutants.

### Sanger sequencing

For wild-type samples, PCR products were purified and sent to outside facilities for Sanger sequencing. For samples with mutations, PCR products were purified and cloned into TA vectors (Takara, Japan). Plasmids extracted from clones that were successfully inserted with target genes were sent for Sanger sequencing.

### Statistical analysis

Event-free survival was defined as the time elapsed from study enrollment to induction failure, relapse, or death. Overall survival (OS) was defined as the time from study enrollment until death from any cause. EFS and OS were calculated using the Kaplan–Meier method, and the log-rank test was used for comparisons of Kaplan–Meier curves. All *p*-values were two-sided and *p* < 0.05 was considered as statistically significant. Statistical calculations were performed using SAS version 9.2 (SAS Institute Inc., Cary, NC, USA).

## Results

### HRM analysis

**Figure 1:**
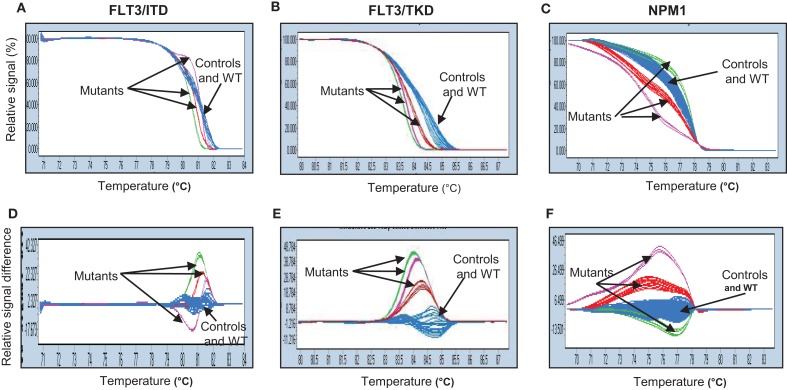
**Genetic variation of *FLT3-ITD* (A,D), *FLT3-TKD* (B,E), and *NPM1* (C,F) genes analyzed by high-resolution melting (HRM) curve analysis**. Fragments of three genes amplified by PCR and HRM analysis from cDNA isolated from patients and normal BM samples were shown in two different plots: normalized plot **(A–C)** and difference plot **(D–F)**. Both plots enable differentiation between control and wild-type samples (blue) and mutants (red, purple, and green). WT, wild-type.

Results were analyzed using the LightCycler^®^ 480 associated software (release 1.5.0). Data were presented in two formats: the normalized plot (Figures 1A–C), in which the amount of intercalating dye remaining at any temperature point was expressed as a fraction of the amount prior to data acquisition; and a difference plot (Figures 1D–F), where the average HRM profile of the control samples was used by the genotype function of the machine software as the standard wild-type profile for subsequent comparison to each of the test samples. Each mutant allele had its own distinctive melting curve when compared to the wild-type allele. The distinct melting curves of the mutant became more apparent when data were represented in a difference plot format than in a normalized plot.

To assess the specificity of the HRM test, we selected nine samples, seven of which tested positive and two negative for *FLT3-ITD*. Direct Sanger sequencing confirmed 100% consistency. Furthermore, we performed similar validation for the *NPM1* gene mutation in six mutants and five wild-type samples. Results were confirmed with 100% consistency when compared to Sanger sequencing (Table 1). Sanger sequencing verification showed that in patients with *FLT3-ITD*, the size of internal tandem duplication varied from 3 to 66 bp. No difference was observed between the size of the ITD and the results seen in HRM analysis in all the samples analyzed. Sequencing of samples with *FLT3-TKD* confirmed the most frequent D835Y mutation. The NPM1 mutation involved 4 bp insertions, which altered the tryptophan at amino acid position 288.

**Table 1:** **Sanger sequencing validation of *FLT3-ITD* (A) and *NPM1* (B) gene mutations detected by HRM analysis using fluorescent dye SYTO-82**.

	No. 1	No. 2	No. 3	No. 4	No. 5	No. 6	No. 7	No. 8	No. 9	Confirmation
**(A) *FLT3-ITD* MUTATION VALIDATION**											
HRM	Mut	Mut	Mut	Mut	Mut	Mut	Mut	Wt	Wt	100%
Sequencing	Mut	Mut	Mut	Mut	Mut	Mut	Mut	Wt	Wt	/

	**No. 1**	**No. 2**	**No. 3**	**No. 4**	**No. 5**	**No. 6**	**No. 7**	**No. 8**	**No. 9**	**No. 10**	**Confirmation**

**(B) *NPM1* MUTATION VALIDATION**											
HRM	Mut	Mut	Mut	Mut	Mut	Mut	Wt	Wt	Wt	Wt	100%
Sequencing	Mut	Mut	Mut	Mut	Mut	Mut	Wt	Wt	Wt	Wt	/

**Figure 2:**
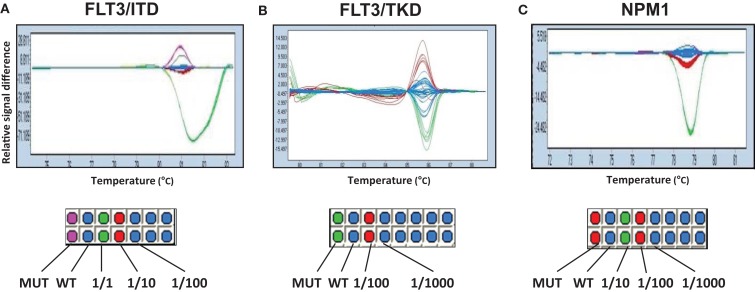
**Evaluation of the ability of HRM for MRD detection**. Mutant cDNA of *FLT3-ITD* **(A)**, *FLT3-TKD* **(B)**, and *NPM1* **(C)** genes was titrated and mixed with different ratio of wild-type cDNA to produce a range of mutant allele dilutions. Fractions show the proportion of mutated/wild-type genes. Color differences demonstrate the identification of sample types. Duplications were made in columns.

We also tested whether HRM analysis could be used as a method for MRD monitoring. The ability to detect low levels of *FLT3-ITD*, *FLT3-TKD*, and *NPM1* gene mutations in a background of non-mutated DNA was evaluated by titrating each of the mutant alleles with wild-type DNA to produce a range of the mutant allele dilutions. Unfortunately, the lower limit of detection was 1/10 ~ 1/100 for *FLT3-ITD* and 1/100 ~ 1/1000 for both *FLT3-TKD* and *NPM1*. This suggested that HRM analysis was not sensitive enough for MRD monitoring in this study (Figure 2).

### Patient characteristics and genetic findings

Patient characteristics were summarized in Table 2. The mean age of patients was 7 years (range 0.3–16.9 years). Slightly more males than females were included in the study. Five patients had M3 morphology. However, they were all negative for *t*(15;17) and PML-RARA. Therefore, they were all treated with AML-XH-99 protocol and were included in the analysis. Among all 99 PML-RARA negative AML patients, 22 (24.2%) were positive for RUNX1-RUNX1T1, and 1 (1.0%) was positive for CBFB–MYH11. Fifty four patients (54.5%) had normal karyotype and were defined as CN-AML.

**Table 2:** **Clinical characteristics of 99 AML patients**.

Characteristics	Total patients
Number	99
Mean age (year)	7.0 (0.3–16.9)
Sex, number of patients (%)
Male	65 (65.7)
Female	34 (34.3)
Median WBC count, ×10^9^/L (range)	20 (1.3–262)
Median BM blasts, % (range)	72 (18.4–99.6)
Fab subtype, number of patients (%)
M0	0 (0)
M1	10 (10.1)
M2	47 (47.5)
M3	5 (5.1)
M4	10 (10.1)
M5	23 (23.2)
M6	1 (1.0)
M7	3 (3.0)
Cytogenetic alterations, no. (%)
*t*(8;21)(q22;q22)/RUNX1-RUNX1T1^a^	22 (22.2)
Inv(16)(p13.1q22)/CBFB-MYH11^a^	1 (1.0)
Others^b^	22 (22.2)

*^a^*t*(8;21)(q22;q22) and Inv(16)(q13.1q22) were detected by karyotyping or PCR (for RUNX1-RUNX1T1 and CBFB-MYH11)*.

*^b^Samples with other cytogenetic abnormalities*.

Of the total 99 patients, HRM analysis detected 33 patients with *FLT3-ITD* mutations, 10 with *FLT3-TKD* mutations, and 21 with *NPM1* mutations. Four patients were positive for *MLL-PTD* mutations (Table 3) by RQ-PCR. The presence of the mutations was not mutually exclusive. Among the 33 patients with the *FLT3-ITD* mutations, 4 had *NPM1* mutations, and 2 patients had the *MLL-PTD* mutations. Similarly, among the 10 patients with the *FLT3-TKD* mutations, 2 of them had *NPM1* mutations and 1 had the *MLL-PTD* mutation. One patient was found to have concurrent *FLT3-ITD*, *FLT3-TKD*, and *NPM1* mutations. In total, 66% patients had at least one gene mutation.

**Table 3:** **Mutation frequencies in pediatric AML**.

Gene mutations	Total (*n* = 99)	CN-AML (*n* = 54)
FLT3-ITD	33 (33.3%)	18 (33.3%)
FLT3-TKD	10 (10.1%)	4 (7.4%)
NPM1	21 (21.2%)	13 (24.1%)
MLL-PTD	4 (4%)	3 (5.5%)

Of the 54 CN-AML patients, there were 18 (33.3%) *FLT3-ITD* mutants, 4 (7.4%) *FLT3-TKD* mutants, 13 (24.1%) *NPM1* mutants, and 3 (5.56%) *MLL-PTD* mutants (Table 3).

### Clinical outcome

**Figure 3:**
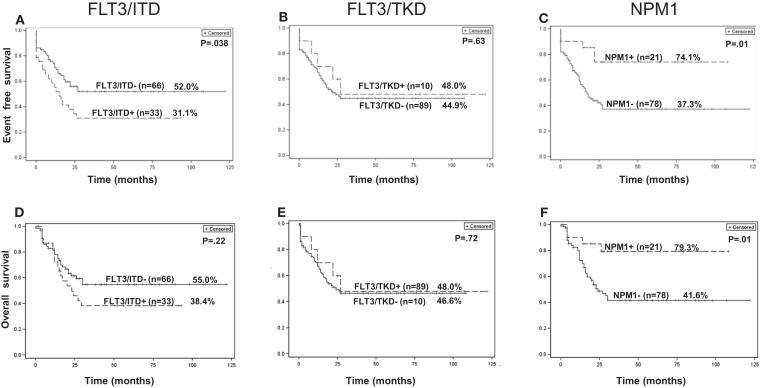
**Kaplan–Meier curves of EFS (A–C) and OS (D–F) for all 99 patients with or without *FLT3-ITD*, *FLT3-TKD*, and *NPM1* gene mutations**.

The EFS and OS at the median follow-up time of 49.6 months for all patients were 45.2 and 50.0%, respectively. The EFS of patients with *FLT3-ITD* mutations were significantly worse when compared to that of patients without *FLT3-ITD* mutations (*p* = 0.038, Figure 3A). The OS for patients with and without *FLT3-ITD* mutations were 38 and 55%, respectively. However, the difference was not statistically significant (Figure 3D). There was no significant difference in EFS and OS for patients with or without *FLT3-TKD* mutations (Figures 3B,E). The EFS and OS for all patients with *NPM1* mutations were significantly better than patients without *NPM1* mutations (*p* = 0.01, Figures 3C,F). All four patients with *MLL-PTD* mutations died from disease.

In the CN-AML group of 54 patients, the EFS and OS time were 50 and 53%, respectively. We found no significant difference in EFS and OS for patients with or without *FLT3-ITD* mutations (Figures S1A,D in Supplementary Material) or *FLT3-TKD* mutations (Figures S1B,E in Supplementary Material). There is a trend toward better EFS in patients with the *NPM1* mutation when compared to patients without the mutation (*p* = 0.07, Figure S1C in Supplementary Material); OS was significantly better (*p* = 0.05, Figure S1F in Supplementary Material). All three patients with *MLL-PTD* mutations in the CN-AML group died of disease progression.

## Discussion

In this study, we developed HRM assays for assessment of *FLT3-ITD*, *FLT3-TKD*, and *NPM1* gene mutations. As previously described, HRM allows closed-tube identification of mutations using a real-time PCR machine now common to most labs. Using the LightCycler^®^ 480 Real-Time PCR System, PCR and analysis can be performed in one instrument. There is also less cDNA processing required, compared to non-homogeneous (gradient or gel-based) mutation screening methods. The PCR was easy to set up and the turn-around time was about 24 h. Fluorescent dye SYTO-82 did not inhibit the PCR, did not affect melting temperature, and was accurate for mutation detection. More importantly, it only cost $0.011 USD/test, a fraction of the cost of Sanger sequencing (25–27, 33).

Previously, Tan et al. (34) studied *FLT3* and *NPM1* exon 12 mutations in a cohort of 44 adult patients with normal karyotype AML. HRM was used as a screening tool for confirmatory sequencing. In the pediatric samples we tested, HRM demonstrate 100% accuracy when compared to the gold standard Sanger sequencing in the randomly selected samples. This was consistent with the previous reports using HRM to detect mutations in different cancer types (33, 35–38). Compared to Sanger sequencing, the cost of HRM was lower and turn-around time was shorter (33). It was shown that *FLT3-ITD* with large base pair insertion could be analyzed with agarose gel electrophoresis (39). However, gel electrophoresis will not be able to differentiate mutant from wild-type when inserted fragments are shorter, such as the *NPM1* mutations in our study, in particular, most of which have only 4 bp insertions.

High-resolution melting analysis also had its limitations. HRM can only serve as a screening method and it will not give the exact mutational status. The allelic ratio of *FLT3-ITD* also could not be identified using HRM. Furthermore, as we experienced, this test was not sensitive enough to be used as MRD monitoring. However, HRM is time- and cost-saving, with the major advantage of preventing sample contamination due to the closed tube system. HRM could be a suitable initial clinical screening tool for resource underprivileged countries with large patient populations. Once a mutation is detected, Sanger sequencing could be used as confirmation if necessary. Overall, it can be considered cost-effective in these regions.

Our analysis included 99 children with AML. The frequencies of *FLT-ITD*, *FLT3-TKD*, and *NPM1* identified in this study are similar to previous reports in the pediatric series (4–8, 11, 12). In our whole cohort of patients, *FLT3-ITD* mutations were significantly associated with worse EFS. In patients with *FLT3-TKD*, no significance in clinical outcome was found. These results were also in agreement with other reports (4–8). Similarly, the favorable outcome among patients with the *NPM1* mutation in our patient cohort was consistent with previously published data (8, 11, 12).

Regarding the prognostic significance of gene mutations in the CN-AML group, we and others found that *FLT3-ITD* and *NPM1* were still the most common among the mutated genes examined (4–8, 11, 12). However, we failed to find a significant difference in the outcome between CN-AML patients with and without *FLT3-ITD* mutations, while the survival advantage in patients with *NPM1* mutations remained significant. The discrepancy in the outcome of CN-AML patients with *FLT3-ITD* mutations in our study when compared to published data could be due to some of the patients possibly not having a high *FLT3-ITD* allelic ratio, or small sample size. In addition, some of the CN-AML patients may not be truly “cytogenetic normal” because FISH was not performed in most of the patients due to the high cost of the test ($350 USD per test).

Our study also established an RQ-PCR analysis for *MLL-PTD* and identified that 4% of patients harbored *MLL-PTD* mutations, similar to the published results (13–15). The survival results of this group were extremely poor. All of the patients with *MLL-PTD* mutations died of recurrent or refractory disease. However, the number is too small to make any meaningful conclusions, and further studies are warranted.

In conclusion, HRM is a fast, cost-effective screening diagnostic method for detection of the gene mutations *FLT3-ITD*, *FLT3-TKD*, and *NPM1*, especially in resource underprivileged countries. The frequencies and outcome prediction values of *FLT-ITD*, *FLT3-TKD*, and *NPM1* gene mutations identified in this study were similar to previous reports in other pediatric studies. Further studies are planned using Sanger sequencing to validate the HRM analysis in all patient samples in current pediatric AML trials.

## Author Contributions

Yin Liu wrote the first draft of this paper. Yin Liu, Jingyan Tang, Shuhong Shen, Peter Gaynon, and Weili Sun were responsible for the design and conception of this manuscript. Peter Wakamatsu contributed for statistical analysis. Jingyan Tang, Huiliang Xue, and Jing Chen were responsible for the provision of patients and data acquisition. Yin Liu and Shuhong Shen performed experiments. All authors provided critical review of the manuscript and approval final version submitted.

## Conflict of Interest Statement

The authors declare that the research was conducted in the absence of any commercial or financial relationships that could be construed as a potential conflict of interest. The Review Editor Matthew James Oberley declares that, despite being affiliated to the same institution as authors Peter Wakamatsu, Paul S. Gaynon and Weili Sun the review process was handled objectively and no conflict of interest exists.

## Supplementary Material

The Supplementary Material for this article can be found online at http://www.frontiersin.org/Journal/10.3389/fped.2014.00096/abstract

## Abbreviations

FLT3, FMS-like tyrosine kinase 3; HRM, high-resolution melting curve; ITD, internal tandem duplication; MLL-PTD, partial tandem duplication within the mixed linage leukemia; NPM1, nucleophosmin 1; TKD, tyrosine kinase domain.
