# Supplementary material for: High-Resolution Melting Curve Analysis, a Rapid and Affordable Method for Mutation Analysis in Childhood Acute Myeloid Leukemia
Source: Front Pediatr. 2014 Sep 9;2:96. doi: 10.3389/fped.2014.00096 (PMC4158872; doi:10.3389/fped.2014.00096)
Supplement: Supplementary file 1 [file Image1.PDF]

Supplemental Figure 1. Liu et al.

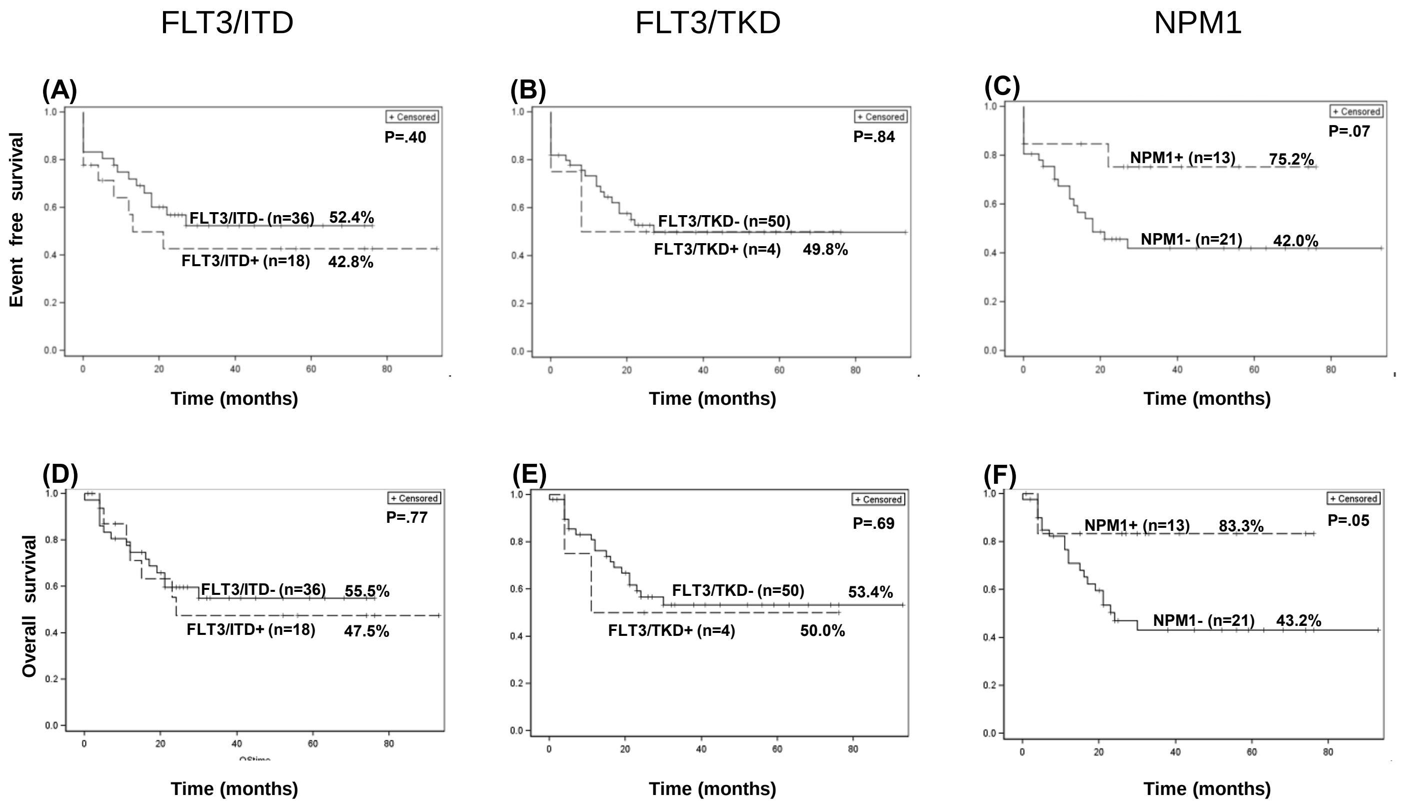

Supplemental Figure 1. Kaplan-Meier curves of EFS (A, B, C) and OS (D, E, F) for patients with CN-AML with or without FLT3-ITD, FLT3-TKD, and NPM1 gene mutations.
